# The Effectiveness of Current Inflammatory Indices and Clinical Scores in Early Diagnosis and Predicting Long-Term Mortality in Patients with Chronic Heart Failure

**DOI:** 10.3390/biomedicines14030539

**Published:** 2026-02-27

**Authors:** Abdulkadir Çakmak, Meryem Çetin, Şirin Çetin

**Affiliations:** 1 Department of Cardiology, Faculty of Medicine, Amasya University, Amasya 05100, Türkiye; 2 Department of Medical Microbiology, Faculty of Medicine, Amasya University, Amasya 05100, Türkiye; meryem.cetin@amasya.edu.tr; 3 Department of Biostatistics, Faculty of Medicine, Amasya University, Amasya 05100, Türkiye; sirin.cetin@amasya.edu.tr

**Keywords:** heart failure, systemic inflammatory response index, Naples prognostic score, inflammatory indices, prognosis, mortality, biomarkers, risk stratification

## Abstract

**Background:** Systemic inflammation through neutrophil-mediated injury, lymphocyte depletion, and monocyte-driven fibrosis plays a central pathophysiological role in heart failure (HF) progression. We investigated the diagnostic and prognostic utility of contemporary inflammatory indices, particularly the Systemic Inflammatory Response Index (SIRI) and Naples Prognostic Score (NPS). **Methods:** This retrospective cohort study enrolled 926 participants (500 HF patients, 426 controls). Multiple inflammatory indices (e.g., SIRI, Prognostic Nutritional Index (PNI)) and prognostic scores (e.g., NPS) were calculated from routine hematological and biochemical parameters. Primary outcomes were HF diagnosis discrimination and 3-month and 24-month all-cause mortality. Receiver operating characteristic (ROC) curve analysis, Kaplan–Meier survival curves, and Cox proportional hazards regression were performed. **Results:** HF patients demonstrated significantly elevated inflammatory burden: SIRI (3.26 vs. 1.06, *p* < 0.001) and NPS (2.00 vs. 1.43, *p* < 0.001). For HF diagnosis, SIRI exhibited superior discriminative performance (AUC = 0.893; 95% confidence interval (CI): 0.871–0.912), substantially exceeding all other indices (*p* < 0.001). For long-term mortality prediction, SIRI maintained the highest accuracy (AUC = 0.677), followed by PNI (AUC = 0.639) and NPS (AUC = 0.613). Kaplan–Meier analysis revealed progressive survival deterioration across NPS categories: 24-month mortality increased from 5.9% (NPS = 0) to 23.0% (NPS = 4), *p* = 0.002. Multivariable Cox regression confirmed independent prognostic value: SIRI >1.86 (HR = 2.232; 95% CI: 1.280–3.892; *p* = 0.005) and NPS > 2 (HR = 1.403; 95% CI: 1.180–1.668; *p* < 0.0001). **Conclusions:** SIRI and NPS represent powerful, readily accessible prognostic tools capturing distinct but complementary pathophysiological domains in HF. These indices offer substantial clinical utility for risk identification and treatment decisions, particularly in resource-limited settings. Future studies should validate these cut-offs and evaluate biomarker-guided therapeutic strategies.

## 1. Introduction

Chronic heart failure (CHF) is recognized as a global public health problem due to its increasing prevalence in developed and developing countries, high mortality rates, and significant economic burden on healthcare systems [1]. Current epidemiological data indicate that more than 64 million individuals worldwide are living with a diagnosis of heart failure (HF), and this number is expected to increase significantly in the coming years with an aging population [2]. According to the European Society of Cardiology guidelines, the 5-year mortality rate in patients diagnosed with CHF exceeds 50%, which is comparable to many malignancies [3].

Inflammation has been shown to play a central role in the development of myocardial dysfunction, ventricular remodeling, and progressive HF [4]. Specifically, it is reported that in patients with heart failure, inflammation contributes to disease progression through mechanisms mediated by endothelial dysfunction and myocardial fibrosis [5].

During the HF process, neutrophils, the primary cells of the acute inflammatory response, exacerbate myocardial damage via the release of reactive oxygen species and proteolytic enzymes. While lymphocytes play a critical role in immune regulation, lymphopenia is recognized as an indicator of poor prognosis in HF patients. Monocytes and their derivative macrophages undertake essential roles in cardiac fibrosis, tissue repair, and remodeling processes. It has been demonstrated that once HF is initiated, neutrophil activation and the release of proinflammatory cytokines are further augmented [6]. The disruption of this cellular equilibrium is one of the fundamental mechanisms reinforcing the progressive nature of HF.

In recent years, inflammatory indices derived from routine complete blood count parameters have gained prominence for the quantitative assessment of systemic inflammation in HF patients. In addition to simple ratios such as the neutrophil-to-lymphocyte ratio (NLR) and monocyte-to-lymphocyte ratio (MLR), more comprehensive indices are being widely utilized in clinical research. The Systemic Immune–Inflammation Index (SII), which integrates neutrophil, platelet, and lymphocyte counts, reflects both inflammatory and thrombotic states. Tang et al. demonstrated that SII is strongly associated with short-term mortality in patients with congestive HF [7]. Similarly, studies based on large-scale population data have revealed significant correlations between inflammatory indices, HF prevalence, and adverse clinical outcomes [8].

The Systemic Inflammation Response Index (SIRI), a more recent index based on a combination of neutrophil, monocyte, and lymphocyte counts, reflects both innate and adaptive immune responses. Wang et al. reported that elevated SIRI values independently predict short- and mid-term mortality risk in elderly HF patients [9]. Recent studies suggest that the prognostic performance of SIRI may exceed that of conventional inflammatory indices such as SII and NLR [10].

The close relationship between inflammation and nutritional status has led to increased clinical attention on conditions like cachexia, which impact prognosis in cardiovascular diseases. Recently, this interplay has been intensively investigated regarding HF prognosis, leading to the continuous development of novel scoring systems. The Glasgow Prognostic Score (GPS) evaluates both inflammation and nutritional status using C-reactive protein (CRP) and albumin levels and has been associated with mortality in CHF [11]. Furthermore, the Naples Prognostic Score (NPS) is a holistic index incorporating albumin, total cholesterol, lymphocyte-to-monocyte ratio (LMR), and NLR, reflecting both inflammatory and nutritional status. Originally defined for solid tumors [12], the NPS has recently garnered attention in HF research. In studies involving patients with decompensated HF, a high NPS has been linked to increased risks of mortality and readmission [13]. A recent study by Aydın et al. demonstrated that NPS is an independent predictor of long-term mortality [14].

Accurate prognostic prediction is of critical importance for risk stratification, determination of treatment intensity, and identification of close follow-up requirements in HF patients. Nutrition-based scores such as the Prognostic Nutritional Index (PNI), another marker emphasized in this field, have also been shown to be associated with cardiovascular mortality [15]. All these data suggest that indices reflecting inflammation and nutritional status may be important clinical tools in HF management.

We planned this study to demonstrate which easily calculated, cost-effective, and rapidly obtainable biomarkers could be prioritized for early risk stratification and prediction of short-term (3-month) and long-term (24-month) mortality in a disease such as HF with high mortality, which significantly impairs quality of life, and to reveal the efficacy of NPS, for which researchers in this field recommend increasing evidence, and association with both HF diagnosis and mortality risk, as well as its prognostic value among clinical and laboratory parameters. By examining the effects of SIRI on mortality in HF patients in addition to NPS specifically, we sought to demonstrate the potential contribution of these simple, inexpensive, and easily applicable parameters in clinical risk stratification in a patient group with a population larger than many studies in the literature.

## 2. Material and Methods

### 2.1. Study Design and Participants

This study was designed as a retrospective, single-center analysis of patients with HF followed at our institution. The study period spanned from 1 May 2023, to 31 May 2025. Patients meeting the diagnostic criteria for HF according to established clinical practice guidelines were included in the study [16]. Complete medical records, including clinical, laboratory, and demographic data, were available for all enrolled patients. The study protocol was approved by the local institutional ethics committee. Due to the retrospective nature of the study, the requirement for individual informed consent was waived by the same ethics committee.

Patients were eligible for inclusion if they fulfilled all of the following criteria including (1) aged 18 years or older (adult patients); (2) hospitalized or managed as outpatients in the cardiology department with a diagnosis of HF; and (3) had accessible and complete demographic, clinical, and laboratory data in their medical records; and also (4) we evaluated hospitalized patients after the acute decompensation period. Blood values were obtained during follow-up in the inpatient ward, when patients were clinically stable in terms of HF, and were analyzed in this study.

Conversely, the exclusion criteria were as follows: patients under 18 years of age; individuals with hematological or other organ malignancies; patients presenting with severe infection (such as sepsis or septic shock); those with chronic inflammatory diseases; patients who underwent major surgery within one month prior to admission; individuals with advanced liver disease, intestinal malabsorption, or nephrotic syndrome; patients with severe burns or trauma; those with a history of myocardial infarction within the last three months; and patients with missing data.

### 2.2. Ethical Statement

The study protocol was approved by the Amasya University Rectorate Non-Interventional Clinical Research Ethics Committee (Approval Date and Document Number: 4 July 2025-272182; Reference Number: E-76988455-050.04-272182; Decision Number: 2025/127). All procedures and experiments were conducted in strict accordance with the relevant guidelines and regulations, specifically the Declaration of Helsinki. Due to the retrospective nature of the study, the requirement for obtaining informed consent from the participants was waived by the Amasya University Rectorate Non-Interventional Clinical Research Ethics Committee.

### 2.3. Data Collection and Definitions

Patient data were retrospectively collected from hospital electronic records and laboratory databases. Demographic and clinical variables recorded for each patient included age, gender, and major comorbidities such as diabetes mellitus (DM), hypertension (HT), cerebrovascular disease (history of stroke or transient ischemic attack (TIA)), and peripheral vascular disease (PVD). Left ventricular ejection fraction (LVEF) was obtained from transthoracic echocardiography data performed during hospital admission or outpatient follow-up. The modified Simpson method was used to calculate LVEF in patients.

The definition of HF (HF with reduced EF, HF with mildly reduced EF, HF with improved EF, or HF with preserved EF) was based on all criteria, including symptoms, physical examination, laboratory tests, and radiological examinations (such as echocardiography, posteroanterior chest X-ray, and computed tomography (CT) scans) obtained from hospital electronic medical records. Patients with HF symptoms and signs, together with supporting laboratory and radiological findings, were included in the HF group, whereas patients without HF symptoms and signs and without supporting laboratory and radiological findings were included in the non-HF group.

Mortality was defined as short-term 3-month and long-term all-cause mortality occurring during a mean 24-month follow-up period. Follow-up mortality data were obtained through verification and confirmation via the patient’s hospital records and the Central Population Management System (MERNIS), where deaths are registered in our country.

Laboratory parameters were compiled from blood samples obtained during hospital admission or outpatient follow-up. Complete blood count results including total white blood cell count (WBC), neutrophil count, lymphocyte count, monocyte count, hemoglobin, and platelet count, as well as blood chemistry and plasma glucose, renal function tests (creatinine, blood urea nitrogen (BUN)), liver enzymes—alanine aminotransferase, aspartate aminotransferase (ALT, AST)—lipid profile (total cholesterol (TC), low-density lipoprotein cholesterol (LDL-C), high-density lipoprotein cholesterol (HDL-C), triglycerides (TG)), and biomarker values such as CRP, serum albumin, and uric acid were recorded. These values were used to calculate various inflammation-related indices and risk scores, including CHA_2_DS_2_-VA score, GPS, NPS, SIRI, CALLY, pan-immune-inflammation value (PIV), NLR, LMR, MLR, SII, PNI, and CAR.

Inflammatory indices and prognostic scores were defined and calculated as follows:

NLR: Neutrophil count (×10^9^/L)/lymphocyte count (×10^9^/L);

LMR: Lymphocyte count (×10^9^/L)/monocyte count (×10^9^/L);

MLR: Monocyte count (×10^9^/L)/lymphocyte count (×10^9^/L);

SII: Platelet count (×10^9^/L) × neutrophil count (×10^9^/L)/lymphocyte count (×10^9^/L);

SIRI: Neutrophil count (×10^9^/L) × monocyte count (×10^9^/L)/lymphocyte count (×10^9^/L);

PNI: Albumin level (g/L) + (0.005 × lymphocyte count (×10^9^/L));

CAR: CRP (mg/L)/albumin (g/L);

PIV: Neutrophil (×10^9^/L) × platelet (×10^9^/L) × monocyte (×10^9^/L)/lymphocyte (×10^9^/L);

CALLY: Albumin (g/L) × lymphocyte count (10^9^/L)/[ CRP (mg/L) × 10];

GPS: 1 point if CRP > 10 mg/L; 1 point if albumin < 35 g/L; 2 points if both conditions are present; otherwise 0 points;

CHA_2_DS_2_-VA score was calculated on a scale of up to eight points with the following components: congestive heart failure or left ventricular systolic dysfunction (C, 1 point); hypertension (H, 1 point); age 75 years and older (2 points); diabetes mellitus (D, 1 point); prior stroke (S, 2 points); vascular disease (V, 1 point); and age 65–74 years (1 point);

NPS: 1 point if total cholesterol (TC) <180 mg/dL, 1 point if albumin <40 g/L, 1 point if NLR > 2.96, and 1 point if LMR ≤ 4.44. 

Otherwise, 0 points were assigned for each parameter. The total score was calculated.

### 2.4. Statistical Method

The sample size for this study was calculated using G*Power version 3.1.9.7 (University of Düsseldorf, Germany) based on an effect size of 0.15, statistical power of 80%, and alpha error of 0.05. A recent study in this field served as the reference for power analysis [17]. The minimum required sample size was determined to be 270 participants.

All statistical analyses were performed using IBM SPSS Statistics version 25.0 (IBM Corp., Armonk, NY, USA). Continuous variables were expressed as mean ± standard deviation (SD) for normally distributed data or as median (minimum–maximum) for non-normally distributed data. Categorical variables were presented as frequencies and percentages (%).

The normality of continuous variables was assessed using the Kolmogorov–Smirnov and Shapiro–Wilk tests. For variables demonstrating normal distribution, parametric tests were employed; for non-normally distributed variables, non-parametric alternatives were utilized.

For comparisons between two independent groups (HF vs. non-HF; survivors vs. non-survivors), Student’s *t*-test was applied for normally distributed continuous variables, while the Mann–Whitney U test was used for non-normally distributed data. For comparisons involving more than two groups (NPS categories 0–4), one-way ANOVA with Duncan and Tamhane post-hoc tests was employed for parametric data, whereas the Kruskal–Wallis test with Dunn’s post-hoc test was used for non-parametric data.

Categorical variables were compared using either the chi-square test or Fisher’s exact test, as appropriate based on expected cell frequencies.

Relationships between variables were evaluated using Pearson’s correlation coefficient for normally distributed data and Spearman’s rank correlation coefficient (rho) for non-normally distributed data.

To evaluate the discriminative performance of inflammatory indices (NLR, SIRI, SII, PIV, LMR, MLR, CAR, CALLY) and prognostic scores (NPS, GPS, PNI) for both HF diagnosis and long-term mortality prediction, ROC curve analyses were performed. For each marker, the area under the curve (AUC) with 95% confidence intervals (CI) was calculated using the DeLong method [18]. Optimal cut-off values were determined using the Youden Index, which maximizes the sum of sensitivity and specificity. Sensitivity, specificity, and their corresponding 95% CIs were reported for each optimal threshold.

Pairwise comparisons of AUC values between different inflammatory indices and prognostic scores were conducted using the non-parametric method for comparing dependent receiver operating characteristic (ROC) curves, as described by DeLong et al. (1988) [18]. This analysis specifically focused on comparing the prognostic performance of NPS with other indices for both HF diagnosis and mortality prediction.

Kaplan–Meier survival curves were constructed to visualize long-term mortality patterns stratified by SIRI (≤1.86 vs. >1.86) and NPS categories (0–4). Survival differences between groups were assessed using the log-rank test.

Cox proportional hazards regression analysis was performed to evaluate the independent prognostic value of SIRI (dichotomized at >1.86) and NPS (dichotomized at >2) for long-term mortality. Hazard ratios (HR) with 95% confidence intervals were calculated for each predictor.

All hypothesis tests were two-tailed, and a *p*-value < 0.05 was considered statistically significant throughout the analyses.

## 3. Results

The study cohort comprised 926 participants, including 500 patients with HF and 426 controls without HF. Baseline demographic analysis revealed no significant age difference between groups (HF: 66.73 ± 12.12 years vs. Non-HF: 62.82 ± 12.98 years, *p* = 0.184). However, the HF group demonstrated a significantly higher proportion of males (77.0% vs. 68.3%, *p* = 0.003), consistent with established epidemiological patterns of HF prevalence (Table 1).

Patients with HF exhibited significantly longer hospitalization durations (5.12 ± 5.52 vs. 3.79 ± 2.33 days, *p* < 0.001), reflecting the greater clinical severity and management complexity of this population. Notably, both 3-month (8.4% vs. 1.4%, *p* < 0.001) and 24-month (17.6% vs. 2.8%, *p* < 0.001) mortality rates were substantially elevated in the HF cohort, underscoring the progressive and life-threatening nature of the condition.

Comorbidity analysis demonstrated significantly higher prevalence of coronary artery disease (54.0% vs. 26.8%, *p* < 0.001), hypertension (72.8% vs. 64.1%, *p* = 0.003), and cerebrovascular accidents (21.8% vs. 6.8%, *p* < 0.001) among HF patients. The diabetes mellitus prevalence, while numerically higher in the HF group (47.4% vs. 42.7%), did not reach statistical significance (*p* = 0.087), suggesting heterogeneity in metabolic risk factor contributions to HF pathogenesis.

Comprehensive laboratory evaluation revealed profound differences in hematological, biochemical, and inflammatory parameters between HF and non-HF groups (Table 2), including how LVEF was markedly reduced in HF patients (42.53 ± 6.73% vs. 59.66 ± 3.75%, *p* < 0.001):

Hematological and Inflammatory Profile: The HF cohort demonstrated significant leukocytosis (10.84 ± 3.93 vs. 9.80 ± 3.27 × 10^9^/L, *p* < 0.001) with elevated neutrophil counts (7.14 ± 3.46 vs. 6.31 ± 2.98 × 10^9^/L, *p* < 0.001) and markedly increased monocyte counts (1.03 ± 0.40 vs. 0.46 ± 0.11 × 10^9^/L, *p* < 0.001), alongside reduced lymphocyte counts (2.11 ± 1.17 vs. 2.52 ± 1.28 × 10^9^/L, *p* < 0.001). These findings indicate robust systemic immune activation and dysregulated inflammatory homeostasis characteristic of chronic HF.

Inflammatory Indices: All calculated inflammatory indices showed significant elevations in HF patients. Most notably, SIRI demonstrated the most striking difference (3.26 [0.20–118.72] vs. 1.06 [0.20–22.82], *p* < 0.001), followed by PIV (1191.57 ± 1776.37 vs. 360.37 ± 381.84, *p* < 0.001) and SII (759.24 vs. 544.91 × 10^9^/L, *p* < 0.001). The NLR and MLR were similarly elevated (*p* < 0.001 for both), while LMR showed the expected inverse relationship (2.09 ± 1.14 vs. 5.89 ± 3.66, *p* < 0.001), reflecting the predominance of monocytes over lymphocytes in HF-associated chronic inflammation.

Prognostic Scores: The NPS was significantly higher in HF patients (2.00 ± 1.18 vs. 1.43 ± 0.96, *p* < 0.001), as were GPS (0.51 ± 0.69 vs. 0.37 ± 0.60, *p* = 0.002) and CHA_2_DS_2_-VA scores (4.04 ± 1.65 vs. 2.52 ± 1.44, *p* < 0.001). Conversely, PNI and CALLY, both inversely related to poor prognosis, were significantly lower in the HF cohort (*p* < 0.001 for both), indicating compromised nutritional and immune status.

Biochemical Markers: HF patients exhibited significantly elevated biomarkers of cardiac stress and organ dysfunction, including higher CRP levels (4.94 vs. 4.00 mg/L, *p* = 0.001), troponin (283 vs. 182 ng/L, *p* = 0.002), glucose (169.72 ± 79.47 vs. 156.72 ± 75.27 mg/dL, *p* < 0.001), BUN (42.91 ± 20.05 vs. 38.20 ± 18.41 mg/dL, *p* < 0.001), creatinine (1.10 ± 0.67 vs. 1.04 ± 0.74 mg/dL, *p* < 0.001), and uric acid (6.01 ± 1.91 vs. 5.68 ± 1.62 mg/dL, *p* = 0.01). Estimated GFR was correspondingly reduced (74.71 ± 23.99 vs. 80.95 ± 23.26 mL/min/1.73 m^2^, *p* < 0.001), confirming the cardiorenal syndrome prevalence in HF. Lower albumin levels (38.20 ± 4.77 vs. 39.38 ± 4.50 g/L, *p* < 0.001) and HDL-C (43 vs. 44 mg/dL, *p* = 0.029) further supported the inflammatory–nutritional derangement paradigm.

Among the 926 study participants, 48 patients (5.2%) experienced mortality within 3 months of enrollment (Table 3). Non-survivors were significantly older (76.23 ± 9.58 vs. 64.31 ± 12.53 years, *p* < 0.001) and demonstrated substantially higher prevalence of coronary artery disease (64.5% vs. 41.4%, *p* < 0.001), emphasizing the prognostic importance of age and atherosclerotic burden:

Cardiac and Risk Stratification: Three-month non-survivors exhibited markedly reduced LVEF (42.67 ± 9.86% vs. 50.84 ± 10.05%, *p* < 0.001) and significantly elevated CHA_2_DS_2_-VA scores (4.96 ± 1.62 vs. 3.25 ± 1.69, *p* < 0.001), confirming the utility of these metrics for short-term risk stratification.

Inflammatory Burden: Profound systemic inflammation characterized the non-survivor phenotype, with dramatically elevated inflammatory indices: SIRI (6.70 ± 7.52 vs. 3.01 ± 4.96, *p* = 0.002), SII (2078.28 ± 2820.13 vs. 924.37 ± 1137.79 × 10^9^/L, *p* = 0.007), PIV (1995.76 ± 2812.51 vs. 744.31 ± 1241.81, *p* = 0.004), and NLR (7.04 ± 7.59 vs. 3.75 ± 4.55, *p* = 0.005). Basal CRP was nearly tripled in non-survivors (41.30 ± 54.39 vs. 14.34 ± 28.81 mg/L, *p* = 0.001), indicating fulminant inflammatory activation as a harbinger of imminent mortality.

Prognostic Scores: All prognostic scores demonstrated significant discriminative capacity. NPS (2.52 ± 1.03 vs. 1.70 ± 1.11, *p* < 0.001), GPS (1.10 ± 0.78 vs. 0.41 ± 0.63, *p* < 0.001), and severely depressed PNI (33.53 ± 5.82 vs. 39.04 ± 4.44, *p* < 0.001) collectively identified patients at highest short-term mortality risk.

During the 24-month follow-up period, 100 patients (10.8%) died, representing a mortality rate approximately double that observed at 3 months. Long-term non-survivors exhibited demographic and clinical profiles similar to short-term mortality victims, though with some notable distinctions reflecting the chronic progressive nature of HF (Table 4).

Demographic and Comorbidity Profile: Non-survivors at 24 months were significantly older (75.18 ± 10.03 vs. 63.69 ± 12.39 years, *p* < 0.001) and demonstrated higher prevalence of coronary artery disease (62.0% vs. 43.1%, *p* < 0.001) and diabetes mellitus (59.0% vs. 43.6%, *p* = 0.04). The emergence of diabetes as a statistically significant predictor in long-term mortality, unlike the 3-month analysis, underscores the cumulative metabolic burden in chronic HF progression.

Cardiac Function and Risk Stratification: LVEF was substantially reduced in non-survivors (41.71 ± 9.61% vs. 51.47 ± 9.75%, *p* < 0.001), while CHA_2_DS_2_-VA scores were markedly elevated (5.53 ± 1.74 vs. 3.08 ± 1.53, *p* < 0.001), demonstrating their robust prognostic utility for long-term outcomes.

Inflammatory and Hematological Parameters: Long-term non-survivors exhibited persistent systemic inflammation, characterized by elevated WBC counts (11.79 ± 5.02 vs. 10.19 ± 3.44 × 10^9^/L, *p* = 0.002), neutrophilia (7.96 ± 4.44 vs. 6.61 ± 3.07 × 10^9^/L, *p* = 0.004), monocytosis (0.98 ± 0.23 vs. 0.74 ± 0.31 × 10^9^/L, *p* < 0.001), and reduced hemoglobin (13.32 ± 2.16 vs. 13.91 ± 2.05 g/dL, *p* = 0.011). The pattern of chronic anemia coupled with leukocytosis reflects ongoing systemic inflammation and bone marrow dysregulation characteristic of advanced HF.

Inflammatory Indices: All inflammatory indices remained significantly elevated in non-survivors: SIRI (5.27 ± 6.28 vs. 2.95 ± 4.98, *p* = 0.001), SII (1444.60 ± 2115.52 vs. 928.44 ± 1156.22 × 10^9^/L, *p* = 0.018), PIV (1416.60 ± 2119.04 vs. 735.64 ± 1259.90, *p* = 0.002), NLR (5.37 ± 6.21 vs. 3.75 ± 4.58, *p* = 0.013), and MLR (0.67 ± 0.90 vs. 0.42 ± 0.37, *p* = 0.006). Notably, the magnitude of SIRI elevation in 24-month mortality (difference: 2.32 units) was more modest than in 3-month mortality (difference: 3.69 units), suggesting that while SIRI remains prognostic, its discriminative power may be strongest for imminent mortality risk.

Biochemical and Nutritional Markers: Long-term non-survivors demonstrated significantly worse renal function (estimated GFR: 63.64 ± 24.77 vs. 79.26 ± 23.18 mL/min/1.73 m^2^, *p* < 0.001), hepatic dysfunction (AST: 66.89 ± 93.19 vs. 41.25 ± 56.92 U/L, *p* = 0.008; ALT: 41.45 ± 69.50 vs. 26.78 ± 26.41 U/L, *p* = 0.039), and metabolic derangement (glucose: 194.80 ± 97.97 vs. 159.98 ± 74.18 mg/dL, *p* = 0.001). The nutritional-inflammatory axis was severely compromised, with lower albumin (36.33 ± 5.82 vs. 39.04 ± 4.44 g/dL, *p* < 0.001), elevated CRP (26.30 ± 43.10 vs. 14.46 ± 29.20 mg/L, *p* = 0.009), and decreased CALLY index (2.60 ± 4.80 vs. 3.70 ± 6.29, *p* = 0.040).

Prognostic Scores: All composite prognostic scores demonstrated significant discriminative capacity for long-term mortality. NPS (2.16 ± 1.15 vs. 1.69 ± 1.10, *p* < 0.001), GPS (0.75 ± 0.77 vs. 0.41 ± 0.63, *p* < 0.001), and PNI (36.34 ± 5.82 vs. 39.05 ± 4.44, *p* < 0.001) collectively identified patients at sustained elevated mortality risk, supporting their clinical utility in long-term prognostication.

To further elucidate the clinical utility of NPS, we performed a comprehensive stratified analysis categorizing the entire study cohort by NPS categories (0–4). This analysis revealed a striking dose–response relationship between NPS and virtually all clinical, laboratory, and prognostic parameters (Table 5):

Mortality and Clinical Burden: The 24-month mortality rate increased progressively across NPS categories: 5.9% (NPS = 0), 8.0% (NPS = 1), 10.8% (NPS = 2), 14.6% (NPS = 3), and 23.0% (NPS = 4) (χ^2^ = 17.37, *p* = 0.002), demonstrating a clear dose–response relationship. Similarly, hospitalization duration increased linearly from 3.52 ± 1.92 days in the lowest risk category to 6.43 ± 7.41 days in the highest (F = 8.199, *p* < 0.001), reflecting escalating disease severity and complication burden.

Age and Cardiovascular Parameters: Mean age increased across NPS categories (59.70 ± 11.40 to 70.43 ± 11.13 years, F = 16.619, *p* < 0.001), while LVEF declined progressively (52.73 ± 9.75% to 41.69 ± 6.90%, F = 21.121, *p* < 0.001). The CHA_2_DS_2_-VA score similarly escalated from 2.61 ± 1.52 to 4.82 ± 1.60 (F = 28.726, *p* < 0.001), confirming that NPS captures cumulative cardiovascular risk burden.

Hematological and Inflammatory Evolution: The hematological profile demonstrated systematic deterioration with increasing NPS. WBC counts rose from 9.09 ± 2.46 to 11.14 ± 4.82 × 10^3^/μL (F = 12.268, *p* < 0.001), while hemoglobin declined from 14.58 ± 1.68 to 12.93 ± 2.31 g/dL (F = 18.448, *p* < 0.001), suggesting progressive anemia of chronic inflammation. Neutrophil counts nearly doubled from lowest to highest NPS categories (5.22 ± 1.70 to 8.75 ± 4.67 × 10^3^/μL, F = 34.342, *p* < 0.001), while lymphocyte counts were more than halved (2.82 ± 0.98 to 1.05 ± 0.36 × 10^3^/μL, F = 60.512, *p* < 0.001), demonstrating severe immunosuppression in advanced disease.

Inflammatory Indices and Exponential Escalation: The inflammatory indices exhibited dramatic escalation with NPS severity. SIRI increased more than 7-fold from NPS = 0 to NPS = 4 (1.38 ± 0.77 to 10.90 ± 15.15, F = 63.667, *p* < 0.001), representing the most pronounced gradient among all parameters. Similarly, NLR increased 5-fold (1.92 ± 0.53 to 10.96 ± 13.38, F = 68.484, *p* < 0.001), while SII and PIV increased approximately 5–6 fold (F = 59.402 and F = 56.413, respectively, both *p* < 0.001). Conversely, protective indices declined with LMR decreasing from 4.87 ± 2.70 to 1.04 ± 0.29 (F = 41.657, *p* < 0.001), and CALLY plummeted from 5.81 ± 8.24 to 0.96 ± 1.98 (F = 17.012, *p* < 0.001).

Biochemical and Nutritional Deterioration: Higher NPS categories were associated with progressive organ dysfunction and nutritional compromise. Albumin decreased linearly from 42.95 ± 2.21 to 33.79 ± 4.41 g/dL (F = 94.093, *p* < 0.001), while CRP increased exponentially from 5.75 ± 9.67 to 46.80 ± 57.36 mg/L (F = 31.078, *p* < 0.001). Renal function declined (estimated GFR: 84.85 ± 19.65 to 68.48 ± 25.09 mL/min/1.73 m^2^, F = 10.678, *p* < 0.001), and hepatic dysfunction worsened (AST: 33.70 ± 41.71 to 79.18 ± 114.29 U/L, F = 8.483, *p* < 0.001).

Lipid Profile and Reverse Epidemiology: A striking pattern of “reverse epidemiology” emerged in lipid metabolism. Total cholesterol declined from 219.10 ± 32.75 to 135.20 ± 33.09 mg/dL (F = 67.651, *p* < 0.001), LDL-C from 149.75 ± 23.79 to 96.36 ± 27.72 mg/dL (F = 49.630, *p* < 0.001), HDL-C from 49.56 ± 9.81 to 38.38 ± 11.08 mg/dL (F = 19.256, *p* < 0.001), and triglycerides from 189.30 ± 119.41 to 106.90 ± 70.48 mg/dL (F = 16.743, *p* < 0.001). This paradoxical inverse relationship between lipid levels and mortality risk reflects advanced cachexia, malnutrition, and chronic inflammatory consumption characteristic of end-stage HF.

Prognostic Scores Correlation: PNI declined systematically from 42.97 ± 2.21 to 33.80 ± 4.41 (F = 94.324, *p* < 0.001), while GPS increased from 0.10 ± 0.31 to 1.13 ± 0.78 (F = 46.322, *p* < 0.001), demonstrating strong concordance among prognostic scoring systems and validating the multidimensional nature of NPS in capturing disease severity.

**Figure 1:**
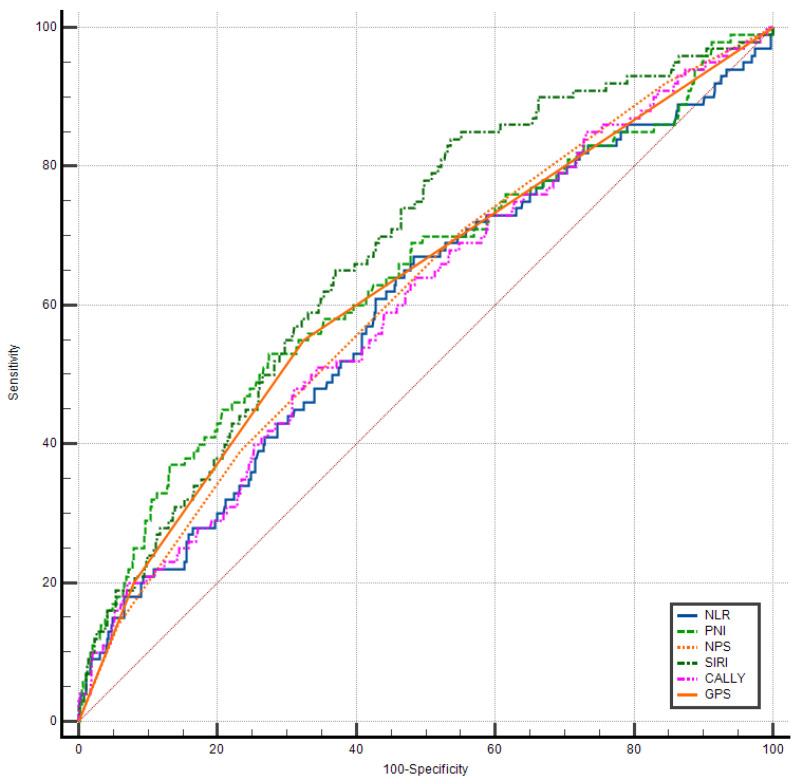
Comparative ROC curve analysis of the prognostic scores and inflammatory indices for HF diagnosis. CALLY: the CRP–albumin–lymphocyte index; GPS: Glasgow prognostic score; NLR: neutrophil-to-lymphocyte ratio; NPS: Naples prognostic score; PNI: prognostic nutritional index; SIRI: systemic inflammatory response index.

ROC curve analysis was performed to evaluate the discriminative performance of inflammatory indices and prognostic scores for HF diagnosis (Table 6, Figure 1). SIRI demonstrated superior diagnostic accuracy with the highest AUC of 0.893 (95% CI: 0.871–0.912, *p* < 0.001), indicating excellent discrimination between HF and non-HF patients. This performance substantially exceeded that of all other biomarkers examined.

NLR demonstrated moderate diagnostic utility (AUC = 0.646, 95% CI: 0.615–0.677), followed by NPS (AUC = 0.638, 95% CI: 0.607–0.669), CALLY (AUC = 0.601, 95% CI: 0.568–0.632), PNI (AUC = 0.577, 95% CI: 0.545–0.609), and GPS (AUC = 0.550, 95% CI: 0.518–0.583). The markedly superior performance of SIRI (AUC difference > 0.24 compared to NLR) suggests that the integrated assessment of neutrophils, monocytes, and lymphocytes captures the complex inflammatory dysregulation in HF more comprehensively than simpler ratios.

Pairwise comparisons using DeLong’s method confirmed that SIRI’s discriminative superiority was statistically significant when compared to all other indices (all *p* < 0.001). The relatively modest performance of traditionally established prognostic scores (GPS, PNI) in HF diagnosis, despite their robust mortality prediction capabilities, suggests that these composite nutritional–inflammatory indices may be more sensitive to chronic disease burden than to the binary presence of HF itself.

Figure 1 displays the comparative ROC curves for all evaluated inflammatory indices and prognostic scores in diagnosing heart failure. The visual representation strikingly demonstrates SIRI’s superior discriminative performance, with its curve positioned substantially above all other biomarkers and approaching the upper left corner of the plot space—the ideal position for a perfect classifier.

The clear separation of SIRI’s curve from the cluster of other biomarkers (NLR, NPS, CALLY, PNI, GPS) visually confirms the quantitative AUC differences and suggests that SIRI captures a unique inflammatory signature specific to HF pathophysiology. The relatively overlapping curves of NLR, NPS, and CALLY indicate comparable but modest diagnostic utility, while PNI and GPS demonstrate discrimination only marginally better than chance (reference diagonal line).

This graphical analysis supports SIRI as a preferred inflammatory biomarker for the risk stratification of HF screening and diagnostic support in clinical practice, while also highlighting the limitations of nutritional–inflammatory composite scores (GPS, PNI) for distinguishing HF from non-HF states.

**Figure 2:**
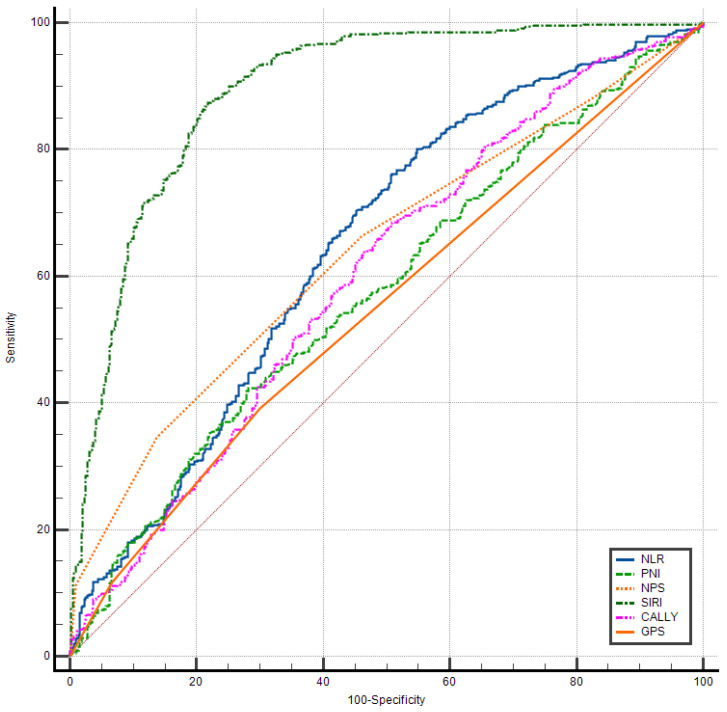
Comparative ROC curve analysis of the prognostic scores and inflammatory indices for predicting long-term mortality. CALLY: the CRP–albumin–lymphocyte index; GPS: Glasgow prognostic score; NLR: neutrophil-to-lymphocyte ratio; NPS: Naples prognostic score; PNI: prognostic nutritional index; SIRI: systemic inflammatory response index.

For long-term (24-month) mortality prediction, the discriminative landscape differed notably from HF diagnosis (Table 7, Figure 2). SIRI maintained the highest predictive accuracy (AUC = 0.677; 95% CI: 0.646–0.707), though its performance was more modest than for HF diagnosis, suggesting that inflammatory burden, while prognostically important, is not the sole determinant of long-term outcomes.

Interestingly, PNI emerged as the second-best predictor (AUC = 0.639; 95% CI: 0.607–0.670), followed closely by GPS (AUC = 0.623; 95% CI: 0.591–0.654), NPS (AUC = 0.613; 95% CI: 0.580–0.644), CALLY (AUC = 0.599; 95% CI: 0.567–0.631), and NLR (AUC = 0.593; 95% CI: 0.561–0.625). The convergence of AUC values across different biomarkers (range: 0.593–0.677) suggests that multiple pathophysiological axes—inflammation, nutrition, and immune dysfunction—contribute comparably to long-term mortality risk.

The relatively superior performance of nutritional indices (PNI, GPS) in association with mortality risk compared to the diagnosis of HF highlights the critical role of nutritional status and chronic inflammatory–catabolic states in determining long-term survival. The modest AUC values (all < 0.70) collectively indicate that while these biomarkers provide meaningful prognostic information, optimal risk stratification likely requires integration with other clinical, imaging, and biomarker data.

**Figure 3:**
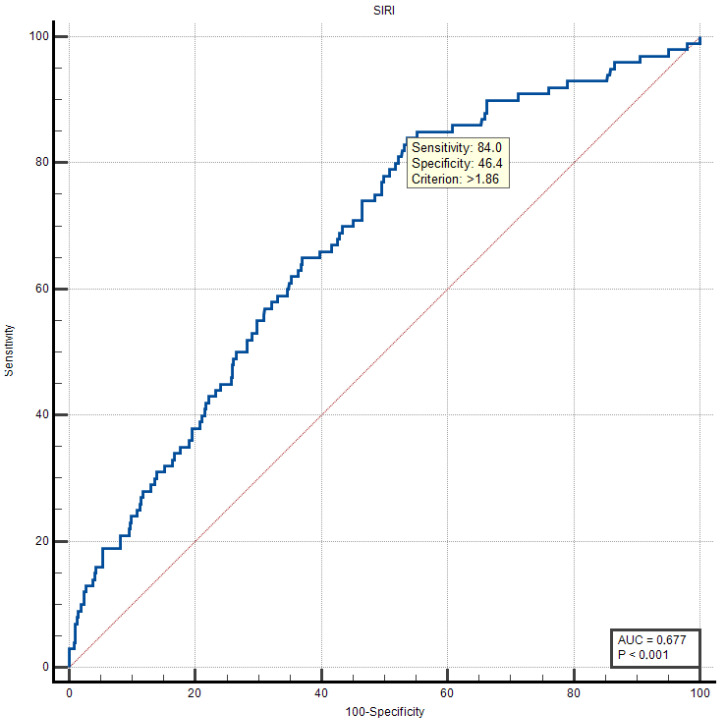
ROC curve analysis and optimum cut-off point value for SIRI in predicting long-term mortality. AUC: area under the curve; SIRI: systemic inflammatory response index.

To optimize clinical applicability of SIRI for mortality risk stratification, we performed Youden Index analysis to determine the optimal cut-off value (Figure 3). The analysis identified SIRI > 1.86 as the optimal threshold, yielding a sensitivity of 84.0% and specificity of 46.37% (Youden Index J = 0.3037).

This relatively low specificity reflects the clinical reality that elevated SIRI is associated with HF risk in patients due to the inherent inflammatory nature of the disease. However, the high sensitivity ensures excellent negative predictive value—patients with SIRI ≤ 1.86 are at substantially lower mortality risk. This characteristic makes SIRI > 1.86 particularly valuable for identifying patients who require intensified monitoring, aggressive pharmacotherapy, and potential consideration for advanced therapies (mechanical circulatory support, transplant evaluation).

The chosen cut-off prioritizes sensitivity over specificity, a clinically appropriate balance given that the consequences of missing high-risk patients (false negatives) substantially outweigh those of over-identifying low-risk patients (false positives) in the HF population.

**Figure 4:**
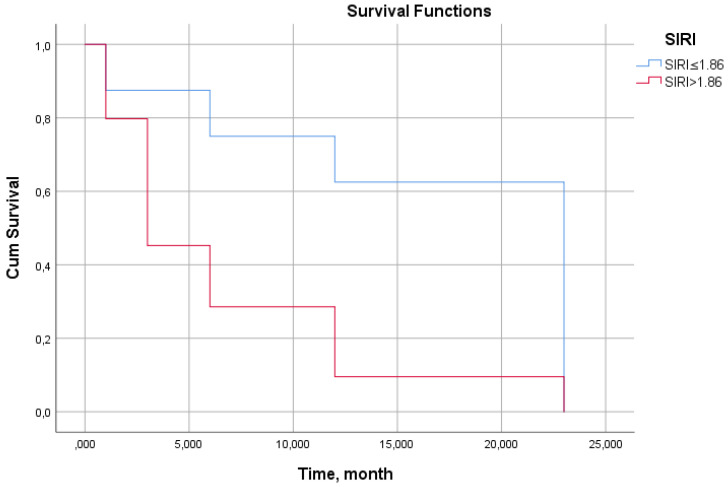
Kaplan–Meier survival curves according to SIRI levels. SIRI: systemic inflammatory response index.

Kaplan–Meier survival analysis stratified by SIRI threshold (≤1.86 vs. >1.86) revealed pronounced and progressive divergence of survival curves over the 24-month follow-up period (Figure 4, log-rank *p* < 0.001). SIRI values >1.86 were associated with a higher mortality risk in patients throughout the entire observation period, with survival probability declining from approximately 92% at 3 months to 78% at 24 months.

In contrast, the SIRI ≤ 1.86 cohort maintained a significantly more favorable survival trajectory, with 24-month survival approximating 95%. The early divergence of curves (evident by 3 months) indicates that SIRI is associated with imminent mortality risk, while the sustained separation throughout follow-up confirms its utility for long-term prognostication.

Cox proportional hazards analysis confirmed that SIRI > 1.86 independently predicted mortality with a hazard ratio of 2.232 (95% CI: 1.280–3.892, *p* = 0.005), indicating that patients exceeding this threshold face more than double the mortality risk compared to those below it, after adjustment for other covariates. This robust association persisted despite the multifactorial nature of HF mortality, emphasizing SIRI’s value as a simple, readily available prognostic tool for the risk stratification.

**Figure 5:**
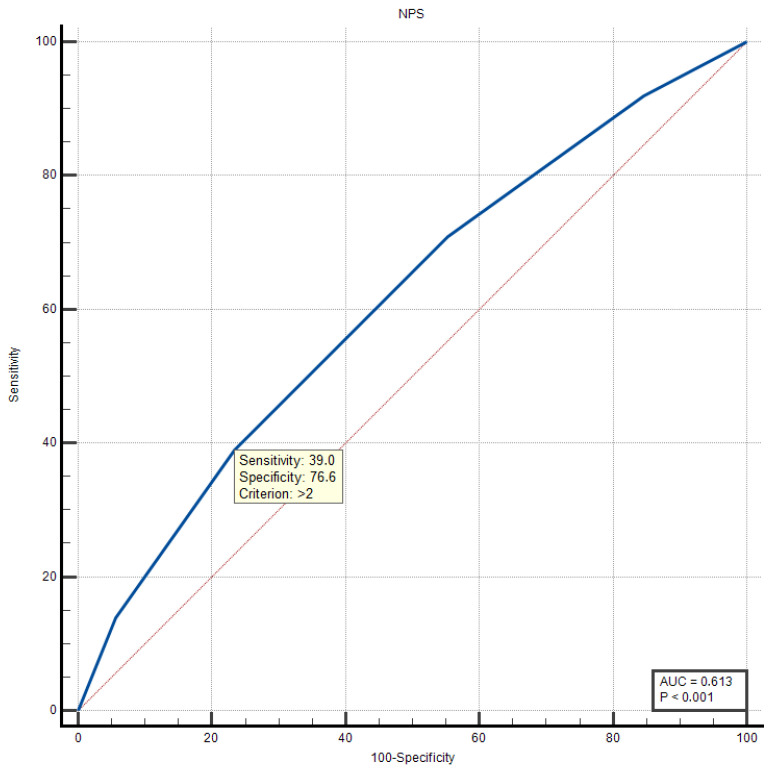
ROC curve analysis and optimum cut-off point value for NPS in predicting long-term mortality. AUC: area under the curve; NPS: Naples prognostic score.

According to the Youden Index analysis, the NPS > 2 as the optimal cut-off value was associated with the mortality risk (Figure 5), demonstrating a more balanced trade-off between sensitivity (39.0%) and specificity (76.63%) compared to SIRI (Youden Index J = 0.1563). This reflects NPS’s different prognostic profile—while less sensitive than SIRI for capturing all at-risk patients, its higher specificity makes it particularly valuable for identifying a subset of patients at especially elevated risk.

The lower sensitivity reflects NPS’s design as a composite score integrating multiple parameters (albumin, cholesterol, NLR, LMR), making it less responsive to isolated inflammatory perturbations but more specific for patients with multidimensional deterioration across nutritional, inflammatory, and immune axes. The NPS > 2 threshold effectively identifies patients with advanced disease characterized by concurrent hypoalbuminemia, hypocholesterolemia, elevated inflammation, and immunosuppression—a constellation particularly associated with poor prognosis.

**Figure 6:**
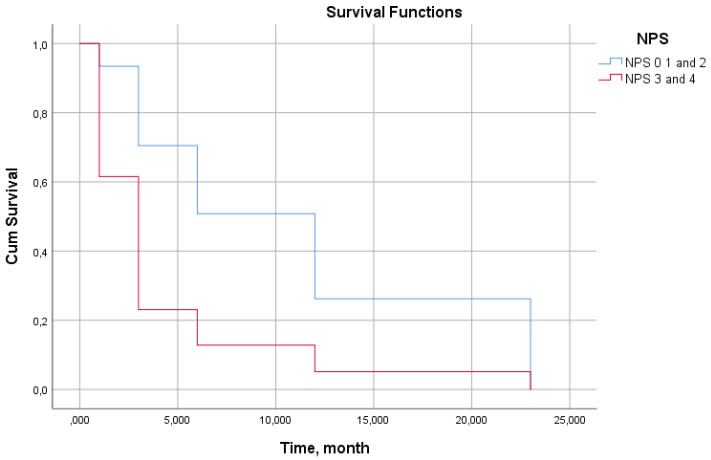
Kaplan–Meier survival curves stratified by NPS. NPS: Naples prognostic score.

Kaplan–Meier analysis stratified by NPS categories (0–4) revealed a clear dose–response relationship between NPS and mortality (Figure 6, log-rank *p* < 0.001). Survival curves demonstrated progressive and sustained separation across all NPS categories, with the most dramatic divergence occurring between NPS = 0–1 (lowest risk) and NPS = 3–4 (highest risk) groups.

The 24-month survival probability declined systematically: approximately 94% for NPS = 0, 92% for NPS = 1, 89% for NPS = 2, 85% for NPS = 3, and 77% for NPS = 4. Notably, an NPS = 3–4 was associated with both an earlier and steeper decline in survival curves and a significant increase in mortality that became apparent at 6 months and then accelerated thereafter.

Cox regression analysis confirmed that NPS > 2 independently was associated with the mortality risk with a hazard ratio of 1.403 (95% CI: 1.180–1.668, *p* < 0.0001), indicating a 40% increase in mortality risk for each point increase in NPS beyond this threshold. The categorical survival analysis visually reinforces the quantitative NPS-mortality dose–response relationship demonstrated in Table 5, validating NPS as a robust stratification tool that integrates inflammatory, nutritional, and immune parameters into a clinically actionable prognostic framework.

Table 8 presents the results of multivariable Cox proportional hazards regression analysis evaluating the independent prognostic value of SIRI and NPS for long-term (24-month) mortality prediction in HF patients. This analysis adjusts for potential confounding variables to isolate the true predictive capacity of these inflammatory and prognostic markers.

The following describes the predictive capacity of these inflammatory and prognostic markers:

SIRI > 1.86 as an Independent Mortality Predictor: SIRI values exceeding 1.86 are associated with a significantly increased mortality risk in patients. This hazard ratio indicates that individuals crossing this threshold face more than double the mortality risk compared to those with lower SIRI values, independent of other clinical covariates. The robust statistical significance (*p* = 0.005) and narrow confidence interval underscore SIRI’s reliability as a prognostic biomarker. From a pathophysiological perspective, this elevated hazard reflects the cumulative burden of neutrophil-driven oxidative injury, monocyte-mediated fibrosis, and lymphocyte depletion—three converging immune–inflammatory axes that accelerate the risk of myocardial dysfunction and systemic decompensation in heart failure.

NPS > 2 as a Continuous Risk Gradient: The NPS demonstrated a different prognostic profile, with each point increase beyond the threshold of two conferring a 40.3% increment in mortality risk. The exceptionally strong statistical significance (*p* < 0.0001) reflects the multidimensional nature of NPS, which integrates nutritional depletion (hypoalbuminemia, hypocholesterolemia), systemic inflammation (elevated NLR), and immunosuppression (depressed LMR). Unlike SIRI’s dichotomous threshold effect, NPS demonstrates a dose–response relationship across its entire range (0–4), making it particularly valuable for granular risk stratification. The lower point estimate (HR = 1.403 vs. 2.232 for SIRI) belies NPS’s clinical utility—while individual increments confer modest risk increases, the cumulative effect across multiple points identifies patients with profound multisystem deterioration associated with a risk of particularly poor prognosis.

Complementary Prognostic Roles: These findings suggest that SIRI and NPS capture distinct but complementary pathophysiological domains. SIRI primarily reflects acute-on-chronic inflammatory burden and immune dysregulation, making it most sensitive to imminent decompensation risk. NPS, by contrast, integrates inflammatory, nutritional, and metabolic axes, providing a more holistic assessment of chronic disease burden and frailty. The statistical independence of both predictors in multivariable modeling indicates they provide additive rather than redundant prognostic information, supporting their combined use for comprehensive risk stratification in clinical practice.

**Figure 7:**
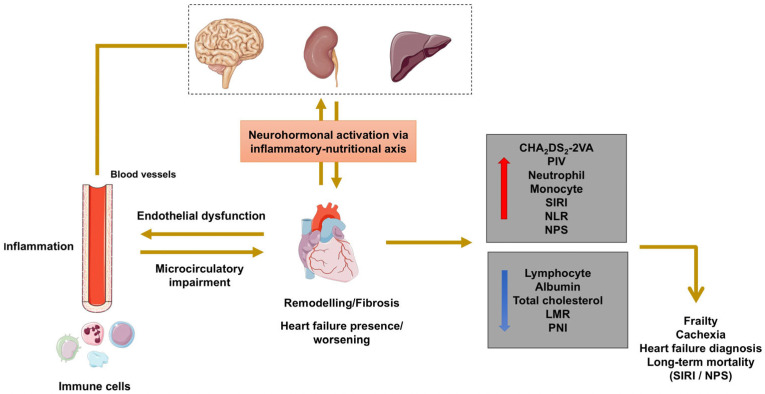
Inflammatory–nutritional axis in HF to reflect the results of this study. Abbreviations: SIRI, systemic inflammatory response index; NPS, Naples prognostic score; PNI, prognostic nutritional index; NLR, neutrophil-to-lymphocyte ratio; LMR, lymphocyte-to-monocyte ratio.

A schematic illustration of the inflammatory–nutritional axis in HF is depicted in Figure 7, reflecting the empirical results of our study.

## 4. Discussion

In this retrospective study, we evaluated 926 patients (500 with HF; 426 without HF) at our hospital. We investigated the efficacy of numerous inflammatory indices and prognostic scores in diagnosing HF and the association with both short-term (3 months) and long-term mortality (24 months). We completed our analyses through comparison of clinical and laboratory variables between groups with and without heart failure, the association with both short- and long-term mortality in this patient cohort, and additional evaluation comparing multiple parameters according to NPS score. While we initially found statistical significance with SIRI, NLR, NPS, and CALLY values regarding the risk of HF development, we revealed that long-term mortality in HF patients demonstrated highly significant correlations primarily with SIRI, PNI, GPS, and NPS values. We demonstrated that each one-point increase in NPS score was significantly associated with HF in a manner indicating unfavorable patterns for variables such as inflammatory indices, length of hospital stay, LVEF, CHA_2_DS_2_-VA, mortality, and GPS.

HF is a highly complex disease. It is a chronic condition with high mortality, characterized by exacerbations and remissions, in which cardiac factors, clinical features, immune system modulation, and inflammation play active roles [8]. In HF, neutrophils, lymphocytes, and monocytes play pivotal roles in the inflammatory process. Neutrophils, which are white blood cells, are associated with the initiation and continuation of the inflammatory process and the immune modulation of the disease. In patients with heart failure, neutrophils become activated and extensively secrete cytokines and oxidative stress molecules. In the pathophysiology of HF, neutrophilia has been independently associated with the severity and prognosis of HF [6]. Lymphocytes, on the other hand, play roles in both adaptive immunity and the regulation of innate immunity. In contrast to the resultant effects of neutrophils, they possess protective and positive regulatory properties in vivo. Lymphocytes reduce cytokine release and inhibit the activation of neutrophils and monocytes. They suppress the inflammatory response, thereby protecting the heart from ischemic and non-ischemic injury [19]. Primarily neutrophils, lymphocytes, monocytes, and platelets are associated with HF, and this process is strongly linked to the proinflammatory roles of these cells.

Monocytes, which also play a critical role in atherosclerosis, are among the determinants of short- and long-term mortality rates in HF patients. Circulating monocytes actively participate in inflammatory processes in the early period following myocardial injury, affecting left ventricular remodeling [20]. Platelets, similar to the first three, play an important role in the pathophysiological process of HF development and progression. Increased platelet count and activity enhance the frequency of cardiovascular events. Activated platelets can contribute to the immuno-inflammatory response by releasing inflammatory cytokines and interacting with other activated cell types. Additionally, platelets increase neutrophil activity, causing damage to viable myocytes, which may augment the severity of cardiac injury [21]. Consequently, neutrophils, lymphocytes, monocytes, and platelets can lead to worsening HF by playing bilateral roles in systemic inflammation, myocardial fibrosis development, cardiac endothelial damage, or cardiac contractile function. Nevertheless, the mechanism of the complex relationship between indices derived from complete blood count and HF continues to be investigated.

The NPS includes albumin, total cholesterol, NLR and LMR among its parameters. NPS therefore reflects both inflammation and malnutrition status and was initially used to predict the prognosis of patients undergoing surgery for colorectal malignancy [22]. In subsequent studies, NPS has been found to be associated with poor outcomes in certain cardiovascular diseases with reduced ejection fraction in HF [13]. In our study, we found that NPS was higher in HF patients compared to non-HF patients and was statistically significantly elevated in both the 3-month and 24-month mortality groups compared to survivors. We observed that long-term survival decreased markedly on the Kaplan–Meier curve in patients with NPS scores of 3 and 4, while Cox regression analysis revealed that patients with NPS scores > 2 had a 1.4-fold-higher long-term mortality compared to those with NPS scores of 0, 1, and 2. These higher NPS scores were associated with an increased risk of long-term mortality, consistent with the results of a recent large-scale study in this field [14]. As a novel finding, in our additional analysis according to all NPS scores, we demonstrated that as NPS score increased, clinical or laboratory parameters showed statistically significant changes between groups in a clinically unfavorable direction.

SIRI is an excellent indicator of chronic inflammation based on neutrophils, lymphocytes, and monocytes. Particularly, SIRI better reflects the long-standing inflammatory state of HF. SIRI, which is essentially a combination of NLR and MLR, has been shown to be a powerful biomarker for assessing inflammation severity in patients with HF and predicting outcomes such as disease diagnosis and mortality [9]. Our findings are consistent with previous research demonstrating significant associations in terms of SIRI’s efficacy in detecting increased HF risk and short- and long-term mortality [10]. In our Cox regression analysis investigating the relationship between SIRI values and long-term mortality, we determined that patients were 2.23-fold more at risk for mortality development at index values > 1.86. In our study, we found that SIRI was the most prominent in terms of prediction compared to other indices and scores with which it was compared. In subgroup analysis with NPS, we demonstrated that SIRI also increased significantly in high-risk groups.

SII is a powerful index for predicting clinical prognosis in HF patients, and elevated SII levels have been associated with worse survival outcomes in patients with chronic HF [23]. Contrary to the aforementioned study, another study demonstrated that SII had no significant association with HF [8]. However, in our study, although SII was not as strong as other inflammatory indices, we found it to be statistically significant and moderately effective in determining the association with both HF diagnosis and short- and long-term mortality. In our study, SII also showed significant increases in high-risk groups categorized by score elevation in NPS subgroup analysis.

NLR represents the balance between innate immunity determined through neutrophil count and acquired immunity determined through lymphocyte count. Lymphopenia accompanying neutrophilia in cardiovascular patients is an indicator of adverse events. In the literature, various studies have reported that higher NLR is associated with higher incidence of adverse cardiac events in these patient groups [24]. In the HF field, increasing evidence has revealed that NLR is associated with both disease risk and high HF mortality [7]. In our study, while we found that NLR had quite high association with HF diagnosis, we demonstrated that it was moderately effective in long-term mortality risk stratification.

The CALLY index represents a novel biomarker incorporating three clinically important parameters: CRP as an indicator of systemic inflammation; albumin as a marker of nutritional status; and lymphocyte count reflecting immune competence. CRP has been independently associated with adverse cardiovascular outcomes and is a strong predictor of HF under inflammatory conditions. Albumin, a marker of systemic nutrition, has shown inverse correlations with mortality in HF patients. Similarly, lymphocyte count is frequently reduced in HF due to chronic immune dysregulation, further associating immune function with disease severity [17].

However, although each component of the CALLY index has been comprehensively examined in HF patients, the predictive value of these components as a single index for short- and long-term mortality in HF patients has not been adequately investigated. In our study, similar to the findings in the aforementioned study, we demonstrated that the CALLY index was associated with HF diagnosis; unlike the findings of that study, we showed that it was also significantly associated with short- and long-term mortality risk. As a novel aspect, in NPS group analyses, we demonstrated that CALLY values decreased as risk increased across groups.

PNI is an index that combines two nutritional markers—serum albumin and total lymphocyte count—and has demonstrated efficacy in assessing the nutritional and inflammatory status of critically ill patients for predicting outcomes such as length of hospital stay, postoperative delirium, and mortality and morbidity in cancer and non-cancer surgical conditions. Although a low PNI score (i.e., poor nutritional status) has been shown to be associated with increased morbidity and mortality in patients with malignancies, its role as an indicator of long-term outcome in cardiovascular diseases remains controversial [15]. In the HF field, a recent subgroup analysis of patients from PARAGON-HF [25] and PARADIGM-HF [26] found higher rates of non-cardiovascular death, all-cause mortality, and all-cause hospitalization in patients with the lowest PNI values, that is, the worst nutritional status [27]. In our study, we demonstrated that PNI value was moderately associated with HF diagnosis and significantly associated with short- and long-term mortality risk. We also found that PNI values were significantly lower in groups where HF risk increased with rising NPS scores.

GPS is calculated from serum CRP and albumin values and provides beneficial short- and long-term prognostic information in patients with cancer, acute decompensated HF, and chronic HF. As the GPS score increases from 0 to 1 and 2, both disease risk and short- and long-term mortality risk increase. This prediction has been found to be significantly higher than the prediction provided by CRP or albumin alone [11].

In our study, we found a moderate-level significant relationship between GPS score and HF risk and a statistically strong significant relationship with short- and long-term mortality. In the analysis between NPS groups, the GPS score also showed an increase in high-risk groups with increasing NPS scores.

In the final analysis of our study, we calculated the odds ratios (ORs) and 95% confidence intervals for each variable associated with inflammatory indices and scores and the effectiveness of these indices in determining their association with HF diagnosis and long-term mortality risks. We performed a ROC curve analysis to assess the sensitivity and specificity of inflammatory markers and scores. We determined the optimal cut-off values for SIRI, NLR, NPS, CALLY, PNI, and GPS using ROC curves. We demonstrated the efficacy of numerous parameters in a large patient group through comparative analyses both for the association with HF diagnosis and with short- and long-term mortality risk. Additionally, we conducted a subgroup analysis in the study group based on the NPS score, which we believe will further increase its clinical importance, and reached interesting results that we believe will make significant contributions to the relevant literature.

### Limitations

There are several limitations to our study that warrant consideration. First, as this was a single-center and retrospective analysis, the generalizability of our findings to broader populations may be limited. Although our study includes HF with preserved EF patients in the HF group, our findings primarily reflect the results of HF with reduced EF and HF with mildly reduced EF patients. Caution should be exercised when generalizing the findings to the HF with preserved EF group. The inflammatory confounding effect of comorbid diseases was not matched between groups. Due to its retrospective design, we were unable to definitively determine whether all patients received optimal medical therapy (angiotensin receptor-neprilysin inhibitors (ARNI), SGLT2 inhibitors, oral/IV diuretics, angiotensin converting enzyme inhibitors/angiotensin Receptor Blockers (ACEi/ARB), evidence-based β-blockers, mineralocorticoid receptor antagonists (MRA), inotropes/vasopressors) as recommended by the most recent clinical guidelines. Furthermore, the longitudinal change in the functional capacity of the patients could not be evaluated. Additionally, the lack of certain data, such as natriuretic peptide levels (BNP, NT-proBNP, MR-proANP), and the reliance on laboratory parameters obtained solely at the time of admission—without comparison to subsequent values during long-term follow-up—represent further limitations of this study.

## 5. Conclusions

This comprehensive investigation demonstrates that contemporary inflammatory indices and prognostic scores provide powerful, clinically actionable tools for both the association with HF diagnosis and mortality risk stratification. The convergent findings from ROC analyses, Kaplan–Meier survival curves, and Cox regression modeling collectively establish that these readily calculated, low-cost biomarkers derived from routine complete blood counts and basic metabolic panels offer substantial clinical utility for early risk identification, treatment intensification decisions, and prognostic counseling in HF populations. Given their accessibility, reproducibility, and strong evidence base, SIRI and NPS warrant incorporation into routine HF risk assessment protocols, particularly in resource-limited settings where advanced biomarkers and imaging modalities may be unavailable. NPS is a score with evidence regarding myocardial ischemia. However, there is insufficient evidence in the literature regarding the association of NPS with the diagnosis or prognosis of HF. Therefore, confirming the association of NPS with both the diagnosis and prognosis of HF using SIRI, which has established evidence, will allow clinicians to integrate it as a guide in clinical practice for identifying low-, medium-, and high-risk HF patients, managing their treatment, and determining their prognosis. Future prospective studies should validate optimal cut-off values across diverse populations and evaluate whether biomarker-guided therapeutic intensification strategies can improve clinical outcomes in high-risk patients identified by these indices.

## Figures and Tables

**Table 1:** Basal demographic and clinical variables among groups.

Variables	Non-HF (n = 426)	HF (n = 500)	*p* Value
Age (years)	62.82 ± 12.98	66.73 ± 12.12	0.184
Male, n (%)	291 (68.30)	385 (77.00)	0.003
Hospitalized day	3.79 ± 2.33	5.12 ± 5.52	<0.001
Mortality, n (%), 3 mos	6 (1.40)	42 (8.40)	<0.001
Mortality, n(%), 24 mos	12 (2.81)	88 (17.60)	<0.001
CAD, n (%)	114 (26.80)	270 (54.00)	<0.001
DM, n (%)	182 (42.72)	237 (47.40)	0.087
HT, n (%)	273 (64.08)	364 (72.80)	0.003
CVA, n (%)	29 (6.80)	109 (21.80)	<0.001

CAD: coronary artery disease; CVA: cerebrovascular accident; DM: diabetes mellitus; HF: heart failure; HT: hypertension; mos: months.

**Table 2:** Basic laboratory and inflammatory biomarkers of the patients.

Variables	Non-HF (n = 426)	HF (n = 500)	*p* Value
HGB (g/dL)	13.93 ± 2.08	13.77 ± 2.05	0.223
WBC count (×10^9^/L)	9.80 ± 3.27	10.84 ± 3.93	<0.001
Platelet count (×10^9^/L)	244.84 ± 71.42	245.30 ± 73.69	0.885
Neutrophil count (×10^9^/L)	6.31 ± 2.98	7.14 ± 3.46	<0.001
Lymphocyte count (×10^9^/L)	2.52 ± 1.28	2.11 ± 1.17	<0.001
Monocyte count (×10^9^/L)	0.46 ± 0.11	1.03 ± 0.40	<0.001
Glucose (mg/dL)	156.72 ± 75.27	169.72 ± 79.47	<0.001
HbA_1c_ (%)	6.70 ± 1.85	6.76 ± 1.63	0.596
BUN (mg/dL)	38.20 ± 18.41	42.91 ± 20.05	<0.001
Creatinine (mg/dL)	1.04 ± 0.74	1.10 ± 0.67	<0.001
Estimated GFR (mL/min/1.73 m^2^)	80.95 ± 23.26	74.71 ± 23.99	<0.001
AST (U/L)	36.16 ± 42.31	50.71 ± 74.66	0.11
ALT (U/L)	25.75 ± 24.39	30.60 ± 40.40	0.82
LVEF (%)	59.66 ± 3.75	42.53 ± 6.73	<0.001
CRP (mg/L)	4.00 (0.17–199.00)	4.94 (0.15–200)	0.001
Troponin (ng/L)	182 (16–29,000)	283 (28–50,000)	0.002
Total protein (g/L)	68.69 ± 9.56	67.67 ± 9.40	0.126
Albumin (g/L)	39.38 ± 4.50	38.20 ± 4.77	<0.001
TG (mg/dL)	122 (30–800)	105 (30–800)	<0.001
TC (mg/dL)	177 (62–395)	172 (1–359)	0.327
HDL-C (mg/dL)	44 (21–95)	43 (20–92)	0.029
LDL-C (mg/dL)	120 (50–311)	118 (50–273)	0.903
Uric acid (mg/dL)	5.68 ± 1.62	6.01 ± 1.91	0.01
NLR	3.18 ± 3.05	4.55 ± 5.83	<0.001
CAR	0.10 (0.01–5.20)	0.12 (0.001–8.30)	<0.001
SII (×10^9^/L)	544.91 (84.55–6996.00)	759.24 (18.79–23,843.76)	<0.001
SIRI (×10^9^/L)	1.06 (0.20–22.82)	3.26 (0.20–118.72)	<0.001
LMR	5.89 ± 3.66	2.09 ± 1.14	<0.001
MLR	0.23 ± 0.14	0.63 ± 0.55	<0.001
PIV	360.37 ± 381.84	1191.57 ± 1776.37	<0.001
PNI	39.39 ± 4.50	38.21 ± 4.77	<0.001
GPS	0.37 ± 0.60	0.51 ± 0.69	0.002
NPS	1.43 ± 0.96	2.00 ± 1.18	<0.001
CHA_2_DS_2_-VA score	2.52 ± 1.44	4.04 ± 1.65	<0.001
CALLY	4.27 ± 5.95	2.98 ± 6.27	0.001

AST: aspartate aminotransferase; BUN: blood urea nitrogen; CALLY: the CRP–albumin–lymphocyte index; CAR: C-reactive protein-to-albumin ratio; CRP: C-reactive protein; GFR: glomerular filtration rate; GPS: Glasgow prognostic score; HbA_1c_: glycated hemoglobin; HDL-C: high-density lipoprotein cholesterol; HF: heart failure; HGB: hemoglobin; LDL-C: low-density lipoprotein cholesterol; LMR: lymphocyte-to-monocyte ratio; LVEF: left ventricular ejection fraction; MLR: monocyte-to-lymphocyte ratio; NLR: neutrophil-to-lymphocyte ratio; NPS: Naples prognostic score; PIV: pan-immune-inflammation value; PNI: prognostic nutritional index; SII: systemic immune–inflammation index; SIRI: systemic inflammatory response index; TC: total cholesterol; TG: triglycerides; WBC: white blood cell count.

**Table 3:** Comparison between survivors and non-survivors (3 months).

Parameters	Survivors (n = 878)	Non-Survivors (n = 48)	*p* Value
	Mean ± SD	Mean ± SD	
Demographic and Clinical Features			
Age (years)	64.31 ± 12.53	76.23 ± 9.58	<0.001
Hospitalized day	4.38 ± 3.73	6.90 ± 10.73	0.112
Male, n (%)	644 (73.3)	32 (66.7)	0.318
CAD, n (%)	364 (41.4)	31 (64.5)	<0.001
DM, n (%)	388 (44.2)	31 (64.6)	0.07
HT, n (%)	609 (69.4)	28 (58.3)	0.112
CVA, n (%)	130 (14.8)	4 (8.3)	0.292
Cardiovascular Parameters			
LVEF (%)	50.84 ± 10.05	42.67 ± 9.86	<0.001
CHA_2_DS_2_-VA score	3.25 ± 1.69	4.96 ± 1.62	<0.001
Hematological Parameters			
WBC (×10^9^/L)	10.18 ± 3.48	13.73 ± 5.21	<0.001
HGB (g/dL)	13.91 ± 2.04	12.66 ± 2.23	<0.001
Platelet count (×10^9^/L)	244.72 ± 71.36	251.85 ± 93.25	0.604
Neutrophil count (×10^9^/L)	6.60 ± 3.05	9.69 ± 5.34	<0.001
Lymphocyte count (×10^9^/L)	2.31 ± 1.22	2.21 ± 1.57	0.679
Monocyte count (×10^9^/L)	0.75 ± 0.31	0.94 ± 0.25	<0.001
Biochemical Parameters			
Glucose (mg/dL)	159.85 ± 73.97	234.79 ± 107.53	<0.001
HbA_1c_ (%)	6.71 ± 1.74	7.07 ± 1.50	0.120
BUN (mg/dL)	39.73 ± 18.39	59.37 ± 30.85	<0.001
Creatinine (mg/dL)	1.06 ± 0.70	1.33 ± 0.63	0.006
Estimated GFR (mL/dk/1.73 m^2^)	78.67 ± 23.33	57.55 ± 24.49	<0.001
Total protein (g/dL)	68.48 ± 9.32	61.89 ± 10.33	<0.001
Albumin (g/dL)	39.03 ± 4.44	33.51 ± 5.81	<0.001
AST (U/L)	41.81 ± 57.67	84.40 ± 112.21	0.012
ALT (U/L)	26.76 ± 26.62	57.71 ± 93.15	0.026
Uric acid (mg/dL)	5.79 ± 1.71	6.99 ± 2.65	0.003
TG (mg/dL)	138.72 ± 96.07	121.69 ± 62.64	0.081
TC (mg/dL)	177.88 ± 46.30	170.81 ± 49.55	0.339
HDL-C (mg/dL)	44.74 ± 10.93	44.58 ± 13.81	0.940
LDL-C (mg/dL)	121.49 ± 34.85	112.38 ± 40.42	0.132
Basal CRP (mg/L)	14.34 ± 28.81	41.30 ± 54.39	0.001
Basal troponin (ng/L)	2541.78 ± 5907.21	3853.46 ± 7282.25	0.226
Inflammatory Indexes			
NLR	3.75 ± 4.55	7.04 ± 7.59	0.005
LMR	3.90 ± 3.28	2.59 ± 1.90	<0.001
MLR	0.43 ± 0.45	0.64 ± 0.55	0.014
Basal CAR	0.40 ± 0.86	1.39 ± 1.94	0.001
SII (×10^9^/L)	924.37 ± 1137.79	2078.28 ± 2820.13	0.007
SIRI (×10^9^/L)	3.01 ± 4.96	6.70 ± 7.52	0.002
PIV	744.31 ± 1241.81	1995.76 ± 2812.51	0.004
CALLY	3.64 ± 6.18	2.51 ± 5.72	0.190
Prognostic Scores			
PNI	39.04 ± 4.44	33.53 ± 5.82	<0.001
GPS	0.41 ± 0.63	1.10 ± 0.78	<0.001
NPS	1.70 ± 1.11	2.52 ± 1.03	<0.001

ALT: alanine aminotransferase; AST: aspartate aminotransferase; BUN: blood urea nitrogen; CAD: coronary artery disease; CALLY: the CRP–albumin–lymphocyte index; CAR: C-reactive protein-to-albumin ratio; CRP: C-reactive protein; CVA: cerebrovascular accident; DM: diabetes mellitus; GFR: glomerular filtration rate; GPS: Glasgow prognostic score; HbA_1c_: glycated hemoglobin; HDL-C: high-density lipoprotein cholesterol; HF: heart failure; HGB: hemoglobin; HT: hypertension; LDL-C: low-density lipoprotein cholesterol; LMR: lymphocyte-to-monocyte ratio; LVEF: left ventricular ejection fraction; MLR: monocyte-to-lymphocyte ratio; NLR: neutrophil-to-lymphocyte ratio; NPS: Naples prognostic score; PIV: pan-immune-inflammation value; PNI: prognostic nutritional index; SII: systemic immune–inflammation index; SIRI: systemic inflammatory response index; TC: total cholesterol; TG: triglycerides; WBC: white blood cell count.

**Table 4:** Comparison between survivors and non-survivors (24 months).

Parameters	Survivors (n = 826)	Non-Survivors (n = 100)	*p* Value
	Mean ± SD	Mean ± SD	
Demographic and Clinical Features			
Age (years)	63.69 ± 12.39	75.18 ± 10.03	<0.001
Hospitalized day	4.39 ± 3.83	5.48 ± 7.58	0.160
Male, n (%)	606 (73.4)	70 (70)	0.272
CAD, n (%)	356 (43.1)	62 (62)	<0.001
DM, n (%)	360 (43.6)	59 (59)	0.040
HT, n (%)	563 (68.2)	74 (74)	0.255
CVA, n (%)	125 (15.1)	9 (9)	0.131
Cardiovascular Parameters			
LVEF (%)	51.47 ± 9.75	41.71 ± 9.61	<0.001
CHA_2_DS_2_-VA score	3.08 ± 1.53	5.53 ± 1.74	<0.001
Hematological Parameters			
WBC (×10^9^/L)	10.19 ± 3.44	11.79 ± 5.02	0.002
HGB (g/dL)	13.91 ± 2.05	13.32 ± 2.16	0.011
Platelet count (×10^9^/L)	245.69 ± 69.96	240.12 ± 91.91	0.559
Neutrophil count (×10^9^/L)	6.61 ± 3.07	7.96 ± 4.44	0.004
Lymphocyte count (×10^9^/L)	2.30 ± 1.18	2.29 ± 1.66	0.917
Monocyte count (×10^9^/L)	0.74 ± 0.31	0.98 ± 0.23	<0.001
Biochemical Parameters			
Glucose (mg/dL)	159.98 ± 74.18	194.80 ± 97.97	0.001
HbA_1c_ (%)	6.73 ± 1.77	6.76 ± 1.44	0.841
BUN (mg/dL)	39.46 ± 18.53	51.35 ± 25.21	<0.001
Creatinine (mg/dL)	1.06 ± 0.72	1.21 ± 0.57	0.018
Estimated GFR (mL/dk/1.73 m^2^)	79.26 ± 23.18	63.64 ± 24.77	<0.001
Total protein (g/dL)	68.63 ± 9.39	64.06 ± 9.35	<0.001
Albumin (g/dL)	39.04 ± 4.44	36.33 ± 5.82	<0.001
AST (U/L)	41.25 ± 56.92	66.89 ± 93.19	0.008
ALT (U/L)	26.78 ± 26.41	41.45 ± 69.50	0.039
Uric acid (mg/dL)	5.76 ± 1.71	6.61 ± 2.24	<0.001
TG (mg/dL)	139.92 ± 97.35	120.65 ± 66.70	0.011
TC (mg/dL)	177.90 ± 46.40	174.38 ± 47.15	0.482
HDL-C (mg/dL)	44.57 ± 10.89	46.02 ± 12.62	0.274
LDL-C (mg/dL)	121.51 ± 35.00	116.97 ± 36.70	0.243
Basal CRP (mg/L)	14.46 ± 29.20	26.30 ± 43.10	0.009
Basal troponin (ng/L)	2470.51 ± 5812.54	3760.03 ± 7214.16	0.088
Inflammatory Indexes			
NLR	3.75 ± 4.58	5.37 ± 6.21	0.013
LMR	3.98 ± 3.28	2.66 ± 2.54	<0.001
MLR	0.42 ± 0.37	0.67 ± 0.90	0.006
Basal CAR	0.41 ± 0.87	0.85 ± 1.51	0.005
SII (×10^9^/L)	928.44 ± 1156.22	1444.60 ± 2115.52	0.018
SIRI (×10^9^/L)	2.95 ± 4.98	5.27 ± 6.28	0.001
PIV	735.64 ± 1259.90	1416.60 ± 2119.04	0.002
CALLY index	3.70 ± 6.29	2.60 ± 4.80	0.040
Prognostic Scores			
PNI	39.05 ± 4.44	36.34 ± 5.82	<0.001
GPS	0.41 ± 0.63	0.75 ± 0.77	<0.001
NPS	1.69 ± 1.10	2.16 ± 1.15	<0.001

ALT: alanine aminotransferase; AST: aspartate aminotransferase; BUN: blood urea nitrogen; CAD: coronary artery disease; CALLY: the CRP–albumin–lymphocyte index; CAR: C-reactive protein-to-albumin ratio; CRP: C-reactive protein; CVA: cerebrovascular accident; DM: diabetes mellitus; GFR: glomerular filtration rate; GPS: Glasgow prognostic score; HbA_1c_: glycated hemoglobin; HDL-C: high-density lipoprotein cholesterol; HF: heart failure; HGB: hemoglobin; HT: hypertension; LDL-C: low-density lipoprotein cholesterol; LMR: lymphocyte-to-monocyte ratio; LVEF: left ventricular ejection fraction; MLR: monocyte-to-lymphocyte ratio; NLR: neutrophil-to-lymphocyte ratio; NPS: Naples prognostic score; PIV: pan-immune-inflammation value; PNI: prognostic nutritional index; SII: systemic immune–inflammation index; SIRI: systemic inflammatory response index; TC: total cholesterol; TG: triglycerides; WBC: white blood cell count.

**Table 5:** Comparison of clinical and laboratory parameters according to Naples Prognostic Score.

Parameters	NPS = 0 (n = 135)	NPS = 1 (n = 262)	NPS = 2 (n = 297)	NPS = 3 (n = 171)	NPS = 4 (n = 61)	F/χ^2^	*p* Value
	Mean ± SD	Mean ± SD	Mean ± SD	Mean ± SD	Mean ± SD		
Demographic and Clinical Features							
Gender/male (n, %)	100/74.1	184/70.2	227/76.4	119/69.6	46/75.4	4.072	0.399
Age (years)	59.70 ± 11.40	62.99 ± 11.95	65.32 ± 12.91	69.40 ± 12.61	70.43 ± 11.13	16.619	<0.001
Hospitalized day	3.52 ± 1.92	3.82 ± 2.20	4.66 ± 4.68	5.38 ± 5.82	6.43 ± 7.41	8.199	<0.001
Mortality (24 months) (n, %)	8/5.9	21/8	32/10.8	25/14.6	14/23	17.37	0.002
DM (n, %)	53/39.3	115/43.9	134/45.1	87/50.9	30/49.2	4.718	0.318
HT (n, %)	86/63.7	174/66.4	202/68	121/70.8	54/88.5	13.77	0.008
SVA/TIA (n, %)	32/23.7	64/24.4	32/10.8	3/1.8	3/4.9	60.41	<0.001
Cardiovascular Parameters							
LVEF (%)	52.73 ± 9.75	52.98 ± 10.06	50.26 ± 10.01	48.04 ± 9.82	41.69 ± 6.90	21.121	<0.001
CHA_2_DS_2_-VA score	2.61 ± 1.52	2.93 ± 1.72	3.39 ± 1.69	3.92 ± 1.51	4.82 ± 1.60	28.726	<0.001
Hematological Parameters							
WBC (10^3^/μL)	9.09 ± 2.46	9.84 ± 3.41	10.48 ± 3.38	11.69 ± 4.37	11.14 ± 4.82	12.268	<0.001
HGB (g/dL)	14.58 ± 1.68	14.17 ± 1.83	13.92 ± 1.99	12.94 ± 2.31	12.93 ± 2.31	18.448	<0.001
Platelet count (10^3^/μL)	240.56 ± 64.41	241.09 ± 63.90	238.40 ± 64.59	269.95 ± 91.25	235.10 ± 88.82	6.404	<0.001
Neutrophil count (10^3^/μL)	5.22 ± 1.70	5.89 ± 2.69	6.81 ± 2.85	8.50 ± 3.93	8.75 ± 4.67	34.342	<0.001
Lymphocyte count (10^3^/μL)	2.82 ± 0.98	2.88 ± 1.55	2.19 ± 0.97	1.66 ± 0.69	1.05 ± 0.36	60.512	<0.001
Monocyte count (10^3^/μL)	0.70 ± 0.30	0.70 ± 0.32	0.76 ± 0.30	0.83 ± 0.29	1.02 ± 0.18	18.388	<0.001
Biochemical Parameters							
Glucose (mg/dL)	150.54 ± 69.76	160.72 ± 77.53	161.59 ± 74.18	178.57 ± 85.20	174.75 ± 85.82	3.013	0.017
HbA_1c_ (%)	6.55 ± 1.55	6.69 ± 1.68	6.80 ± 1.86	6.81 ± 1.76	6.76 ± 1.62	0.606	0.658
BUN (mg/dL)	34.01 ± 11.02	36.06 ± 14.15	41.99 ± 20.66	47.59 ± 23.54	50.50 ± 26.92	18.100	<0.001
Creatinine (mg/dL)	0.95 ± 0.35	0.97 ± 0.36	1.10 ± 0.69	1.21 ± 1.00	1.30 ± 1.19	5.850	<0.001
Estimated GFR (mL/dk/1.73 m^2^)	84.85 ± 19.65	81.13 ± 20.69	76.86 ± 24.36	70.86 ± 27.19	68.48 ± 25.09	10.678	<0.001
Total protein (g/dL)	75.12 ± 9.90	70.02 ± 8.91	66.72 ± 8.35	64.61 ± 8.40	61.47 ± 7.97	42.416	<0.001
Albumin (g/dL)	42.95 ± 2.21	40.27 ± 4.09	38.06 ± 3.95	36.05 ± 4.59	33.79 ± 4.41	94.093	<0.001
AST (U/L)	33.70 ± 41.71	34.25 ± 40.08	45.20 ± 57.55	52.54 ± 78.49	79.18 ± 114.29	8.483	<0.001
ALT (U/L)	26.46 ± 21.98	25.89 ± 19.89	28.78 ± 35.88	27.16 ± 37.49	44.57 ± 66.65	4.021	0.003
ALP (U/L)	72.67 ± 22.40	74.51 ± 25.39	80.26 ± 28.84	81.57 ± 30.09	86.93 ± 35.02	5.046	0.001
Uric acid (mg/dL)	5.71 ± 1.47	5.68 ± 1.52	5.85 ± 1.78	6.06 ± 2.04	6.39 ± 2.54	2.795	0.025
Lipid Profile							
TG (mg/dL)	189.30 ± 119.41	147.19 ± 103.28	123.66 ± 72.35	118.55 ± 83.57	106.90 ± 70.48	16.743	<0.001
TC (mg/dL)	219.10 ± 32.75	187.98 ± 45.27	168.85 ± 40.85	158.81 ± 42.40	135.20 ± 33.09	67.651	<0.001
HDL-C (mg/dL)	49.56 ± 9.81	46.92 ± 11.16	43.63 ± 10.55	41.73 ± 10.64	38.38 ± 11.08	19.256	<0.001
LDL-C (mg/dL)	149.75 ± 23.79	127.62 ± 34.96	115.24 ± 32.88	107.05 ± 32.70	96.36 ± 27.72	49.630	<0.001
Inflammation Markers							
Basal CRP (mg/L)	5.75 ± 9.67	7.24 ± 13.10	15.88 ± 29.78	25.34 ± 40.26	46.80 ± 57.36	31.078	<0.001
Basal troponin (ng/L)	645.30 ± 2346.46	1755.37 ± 4992.90	3293.48 ± 6766.50	3508.44 ± 6694.02	4778.95 ± 7619.22	9.212	<0.001
Inflammation Indexes							
NLR	1.92 ± 0.53	2.38 ± 1.73	3.60 ± 2.22	5.92 ± 4.50	10.96 ± 13.38	68.484	<0.001
LMR	4.87 ± 2.70	5.21 ± 4.23	3.53 ± 2.61	2.44 ± 1.73	1.04 ± 0.29	41.657	<0.001
MLR	0.27 ± 0.15	0.29 ± 0.17	0.41 ± 0.23	0.61 ± 0.40	1.22 ± 1.24	81.748	<0.001
Basal CAR	0.13 ± 0.23	0.19 ± 0.38	0.43 ± 0.81	0.78 ± 1.28	1.52 ± 1.97	36.803	<0.001
SII (×10^9^/L)	463.24 ± 191.11	591.53 ± 516.68	860.72 ± 574.19	1610.05 ± 1472.54	2670.10 ± 3432.74	59.402	<0.001
SIRI (×10^9^/L)	1.38 ± 0.77	1.66 ± 1.21	2.71 ± 1.93	5.12 ± 4.93	10.90 ± 15.15	63.667	<0.001
PIV	336.43 ± 225.78	412.06 ± 358.74	648.37 ± 501.54	1401.86 ± 1577.88	2682.63 ± 3881.61	56.413	<0.001
CALLY	5.81 ± 8.24	5.13 ± 6.82	2.63 ± 5.09	2.01 ± 4.52	0.96 ± 1.98	17.012	<0.001
Prognostic Scores							
PNI	42.97 ± 2.21	40.28 ± 4.09	38.07 ± 3.95	36.05 ± 4.59	33.80 ± 4.41	94.324	<0.001
GPS	0.10 ± 0.31	0.26 ± 0.51	0.45 ± 0.61	0.73 ± 0.79	1.13 ± 0.78	46.322	<0.001

ALP: alkaline phosphatase; ALT: alanine aminotransferase; AST: aspartate aminotransferase; BUN: blood urea nitrogen; CAD: coronary artery disease; CALLY: the CRP–albumin–lymphocyte index; CAR: C-reactive protein-to-albumin ratio; CRP: C-reactive protein; CVA: cerebrovascular accident; DM: diabetes mellitus; GFR: glomerular filtration rate; GPS: Glasgow prognostic score; HbA_1c_: glycated hemoglobin; HDL-C: high-density lipoprotein cholesterol; HF: heart failure; HGB: hemoglobin; HT: hypertension; LDL-C: low-density lipoprotein cholesterol; LMR: lymphocyte-to-monocyte ratio; LVEF: left ventricular ejection fraction; MLR: monocyte-to-lymphocyte ratio; NLR: neutrophil-to-lymphocyte ratio; NPS: Naples prognostic score; PIV: pan-immune-inflammation value; PNI: prognostic nutritional index; SII: systemic immune–inflammation index; SIRI: systemic inflammatory response index; TC: total cholesterol; TG: triglycerides; WBC: white blood cell count.

**Table 6:** The effectiveness of inflammatory indices and prognostic scores in predicting heart failure.

Variable	AUC	SE ^a^	95% CI ^b^
SIRI	0.893	0.0109	0.871 to 0.912
NLR	0.646	0.0183	0.615 to 0.677
NPS	0.638	0.0174	0.607 to 0.669
CALLY	0.601	0.0187	0.568 to 0.632
PNI	0.577	0.0187	0.545 to 0.609
GPS	0.550	0.0158	0.518 to 0.583

^a^ DeLong et al., 1988 [18], ^b^ binomial exact. AUC: area under the curve; CALLY: the CRP–albumin–lymphocyte index; CI: confidence interval; GPS: Glasgow prognostic score; NLR: neutrophil-to-lymphocyte ratio; NPS: Naples prognostic score; PNI: prognostic nutritional index; SE: standard error; SIRI: systemic inflammatory response index.

**Table 7:** The effectiveness of inflammatory indices and prognostic scores in predicting long-term mortality for HF patients.

Variable	AUC	SE ^a^	95% CI ^b^
SIRI	0.677	0.0276	0.646 to 0.707
PNI	0.639	0.0322	0.607 to 0.670
GPS	0.623	0.0278	0.591 to 0.654
NPS	0.613	0.0292	0.580 to 0.644
CALLY	0.599	0.0307	0.567 to 0.631
NLR	0.593	0.0316	0.561 to 0.625

^a^ DeLong et al., 1988 [18], ^b^ binomial exact. AUC: area under the curve; CALLY: the CRP–albumin–lymphocyte index; CI: confidence interval; GPS: Glasgow prognostic score; NLR: neutrophil-to-lymphocyte ratio; NPS: Naples prognostic score; PNI: prognostic nutritional index; SE: standard error; SIRI: systemic inflammatory response index.

**Table 8:** Results of Cox regression analysis in predicting long-term mortality for SIRI and NPS values.

Variable	B (β)	SE	Wald	*p*	HR	%95 CI	
						Alt	Üst
SIRI > 1.86	0.803	0.284	8.009	0.005	2.232	1.280	3.892
NPS > 2	0.339	0.088	14.662	<0.0001	1.403	1.180	1.668

B (β): log hazard ratio; CI: confidence interval; HR: hazard ratio; NPS: Naples prognostic score; SE: standard error; SIRI: systemic inflammatory response index.

## Data Availability

The original contributions presented in this study are included in the article. Further inquiries can be directed to the corresponding author.

## Abbreviations

The following abbreviations were used:

ALP: alkaline phosphatase; ALT: alanine aminotransferase; AST: aspartate aminotransferase; AUC: area under the curve; B (β): log hazard ratio; BUN: blood urea nitrogen; CAD: coronary artery disease; CALLY: the CRP–albumin–lymphocyte index; CAR: C-reactive protein-to-albumin ratio; CHA_2_DS_2_-VA: congestive heart failure, hypertension, age, diabetes, stroke/transient ischemic attack, vascular disease; CI: confidence interval; CRP: C-reactive protein; CVA: cerebrovascular accident; DM: diabetes mellitus; GFR: glomerular filtration rate; GPS: Glasgow prognostic score; HbA_1c_: glycated hemoglobin; HDL-C: high-density lipoprotein cholesterol; HF: heart failure; HGB: hemoglobin; HT: hypertension; HR: hazard ratio; LDL-C: low-density lipoprotein cholesterol; LMR: lymphocyte-to-monocyte ratio; LVEF: left ventricular ejection fraction; MLR: monocyte-to-lymphocyte ratio; mos: months; NLR: neutrophil-to-lymphocyte ratio; NPS: Naples prognostic score; PIV: pan-immune-inflammation value; PNI: prognostic nutritional index; SE: standard error; SII: systemic immune–inflammation index; SIRI: systemic inflammatory response index; TC: total cholesterol; TG: triglycerides; WBC: white blood cell count.
